# Experimental Research on Water Droplet Erosion Resistance Characteristics of Turbine Blade Substrate and Strengthened Layers Materials

**DOI:** 10.3390/ma13194286

**Published:** 2020-09-25

**Authors:** Juan Di, Shunsen Wang, Xiaojiang Yan, Xihang Jiang, Jinyi Lian, Zheyuan Zhang, Yonghui Xie

**Affiliations:** 1 College of Mechanical Engineering, Taiyuan University of Science and Technology, No. 66 Waliu Road, Taiyuan 030024, China; juandi@tyust.edu.cn (J.D.); jinyilian@163.com (J.L.); 2 State Key Laboratory of Multiphase Flow in Power Engineering, Institute of Turbomachinery, Xi’an Jiaotong University, No. 28 Xianning West Road, Xi’an 710049, China; yanxjtu@xjtu.edu.cn (X.Y.); zy92zhang@163.com (Z.Z.); yhxie@mail.xjtu.edu.cn (Y.X.); 3 Department of Mechanical Engineering, State University of New York at Stony Brook, New York, NY 11794, USA; xihang.jiang@stonybrook.edu

**Keywords:** water droplet erosion, surface strengthening, 0Cr17Ni4Cu4Nb, stellite alloy, steam turbine

## Abstract

In this paper, the water droplet erosion (WDE) performance of typical martensitic precipitation substrate 0Cr17Ni4Cu4Nb in steam turbine final stage, laser solid solution strengthened sample, laser cladding sample and brazed stellite alloy samples have been studied based on a high-speed rotating waterjet test system. The WDE resistance of several materials from strong to weak is in sequence: Brazed stellite alloy > laser cladding sample > laser solid solution sample > martensitic substrate. Furthermore, the WDE resistance mechanism and the failure mode of brazed stellite alloy have been revealed. It is found that the hard carbide in the stellite alloy is the starting point of crack formation and propagation. Under the continuous droplet impact, cracks grow and connect into networks, resulting in the removal of carbide precipitates and WDE damage. It is proved that the properties of the Co-based material itself is the reason for its excellent WDE resistance. And the carbides have almost no positive contribution to its anti-erodibility. These new findings are of great significance to process methods and parameter selection of steam turbine blade materials and surface strengthened layers.

## 1. Introduction

The last stage blades of thermal power and nuclear power condensing steam turbines often suffer from severe water droplet erosion damage due to the long-term operation in the wet steam zone, which will not only deteriorate the aerodynamic performance of the blades, but also threaten the safe operation of the units [1,2,3,4]. Besides, the lengthened blades in last stage with higher circumferential speed cause more serious erosion damage. Currently, the maximum circumferential velocity of ultra-long turbine blades developed has exceeded 600 m/s [5]. However, the maximum liquid-solid impact velocity is 500 m/s [6,7,8,9] in the former experimental research. Thus, WDE has become one of the most key problems that needs to be solved urgently. At present, application of surface strengthened blade materials in the area prone to water droplet erosion is the most effective method to improve the WDE resistance. The main mechanism is material damage and failure caused by high-speed liquid-solid impact. Many scholars have carried out a lot of research on the WDE and high-speed liquid-solid impact. In terms of experimental research, Smith et al. [10] designed a test device for simulating the water droplet erosion process of the final stage blade material in the final stage environment of the steam turbine. The test chamber was designed as a vacuum environment, considering the working environment of the last stage blade of the steam turbine. And the sample could obtain a higher linear speed by increasing the rotational speed of the motor in the test rig, thereby simulating the process of water droplet impingement of the last stage blades in steam turbine. Hattori et al. [11] carried out liquid-solid impact tests at different angles. In the test, samples of different angles were fixed on the sample holding tool. A high-speed waterjet was sprayed onto the sample through the nozzle. The mass loss of the samples was measured to compare the degree of water droplet erosion damage. The test results indicated that the sample was most severely damaged when the jet vertically impacted the sample and the mass loss rate was proportional to *V*_0_ sin θ. *V*_0_ is the impingement velocity and *θ* is the impact angle. Xie et al. [12] carried out high-speed jet impact test on 1Cr12Ni2W1Mo1V and 1Cr12MoV samples based on the high-speed digital photography system to capture the velocity of the jet. Xu et al. [13,14,15] improved the widely used rotary test rig to change the impact of water droplets or waterjet into the impact of steam flow and added micro-particle thrusters to the test system. Therefore, the influence of micro particles in steam on the water droplet erosion can be carried out based on the brand new test system. Oka et al. [16] studied the WDE characteristics of different ceramic bulk, coatings and metallic materials to analyze the erosion characters and resistance. Thomas [17] explored water droplet erosion characteristics of copper, brass, low carbon steel, silicon steel and alloys under droplets attack through the various surface morphology evolutions in several typical erosion stages based on the rotating jet experimental device. Ahmad et al. [18] studied the droplet impact wear performance of stainless steels and Ti6Al4V at impact velocity within the range of 350–580 m/s. The results showed that the speed exponent *n* for ductile materials was in the range of 3.3–5.

In general, due to the complexity of the water droplet erosion process and the great difference in WDE behavior of different erosion conditions, the effect of the surface strengthened process on WDE performance, the failure mode and WDE resistance mechanism of stellite alloy remain unclear and controversial.

In this paper, the WDE performances of typical martensitic blade substrate and surface strengthened layers samples have been studied experimentally at impact velocity in excess of 600 m/s. The effect of the surface strengthened process on WDE performance and the failure mode of several layers materials have been explored.

## 2. Experimental Procedures

**Figure 1:**
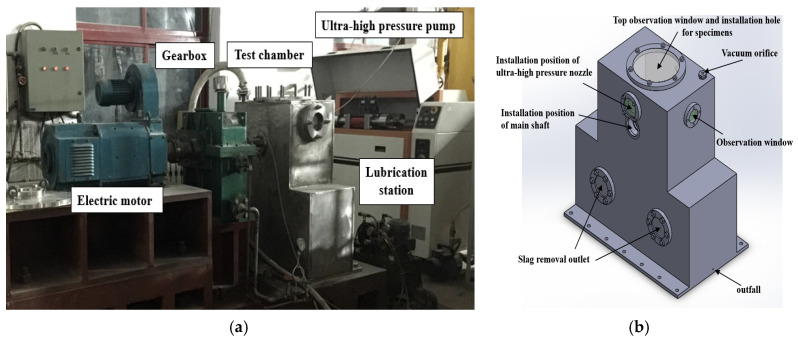
The high-speed waterjet test system. (**a**) Real image of waterjet test system; (**b**). Schematic diagram of the test chamber.

A high-speed waterjet test system has been designed and installed in-house in order to study the water droplet erosion resistance of various materials with impact velocity over 600 m/s. The system is mainly composed of ultra-high pressure water pump, test chamber, DC motor, gear speed increaser, water ring vacuum pump, lubricating oil system and monitoring system. The main part of the test system consists of an ultra-high pressure pump, a rotating shaft, an impeller disk and a test chamber, as shown in Figure 1. Figure 1a,b present a real image of waterjet test system and the schematic diagram of the test chamber, respectively.

The ultra-high pressure pump is used to generate high-speed jet. The core component is a set of ultra-high pressure generator. The maximum stable work pressure of the ultra-high pressure pump is within the range of 240–260 MPa. Under the outlet pressure of 240 MPa, even with a nozzle of 0.3 mm diameter, the jet velocity is above 600 m/s. Based on the adjustable pressure of the ultra-high pressure pump, the jet velocity of the test system could meet a variety of test requirements, which is capable of simulating the high-speed impact process of the condensed droplets under the actual operation condition to the maximal degree compared with the reported water droplet erosion test platforms.

The DC motor is controlled by the motor control cabinet. And the output end of the motor is connected to the gearbox. A coupling is used to connect the output shaft of the gearbox and the rotating shaft. There is an impeller disk installed on the rotating shaft, which drives the specimens to rotate at high speed. A sealed shell is equipped on the outside. A nozzle is applied to lead the water from the ultra-high pressure pump outside the shell, and its position greatly corresponds to the position of the specimen. The shell is also connected with a water ring vacuum pump. A drain pump is arranged at the bottom of the cylinder.

## 3. Experimental Parameters and Results

### 3.1. Test Condition and Parameters

**Figure 2:**
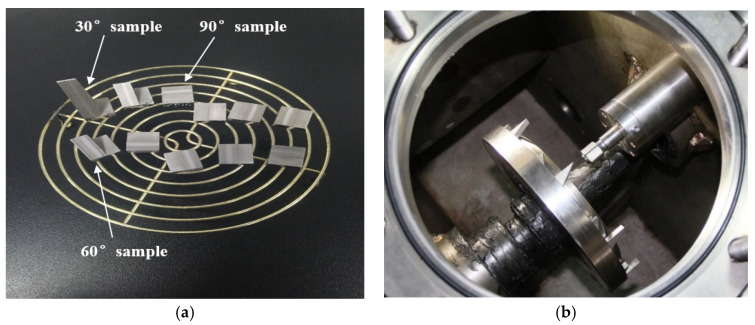
Sample structure and layout. (**a**) The sample structure; (**b**) The layout of samples in impeller disk.

In order to compare the WDE resistance characteristics of different materials, the water droplet erosion characteristics of 0Cr17Ni4Cu4Nb substrate and surface strengthening layers samples are investigated using a high-speed waterjet test system. A VHX-600 3D ultra-depth microscope (Keyence, Osaka, Japan) was used to analyze the eroded morphology of each specimen. The mass of the sample before and after the erosion was measured by the precision electronic balance CPA225D produced by Sartorius (Goettingen, Germany), with a measuring range of 100 g and an accuracy of 0.1 mg. In order to improve the measurement accuracy as much as possible, in addition to leveling, calibrating and peeling before measurement, each sample is demagnetized before testing and washed three times with an acetone or alcohol solution in an ultrasonic cleaner. The test scheme is shown in Table 1. Sample structure and layout diagram are presented in Figure 2.

### 3.2. Test Results

The laser solid solution reinforcement is a strengthening treatment on the surface of the substrate. Laser cladding is to add cladding powder materials to form cladding layer on the surface of the substrate. In order to save the cost, the brazing stellite alloy sample is made by embedding a stellite alloy plate into the matrix. The test duration was 390 min for these samples.

**Figure 3:**
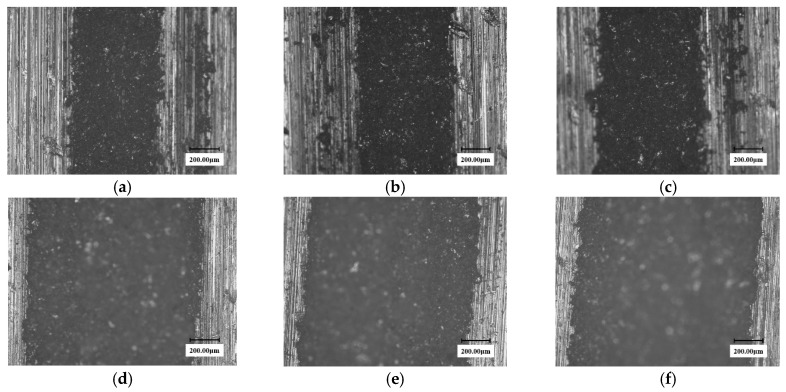
The microscopic morphology of the 90° substrate samples at different testing times in the erosion zone. (nozzle diameter: 0.2 mm). (**a**) 60 min; (**b**) 120 min; (**c**) 150 min; (**d**) 270 min; (**e**) 330 min; (**f**) 390 min.

**Figure 4:**
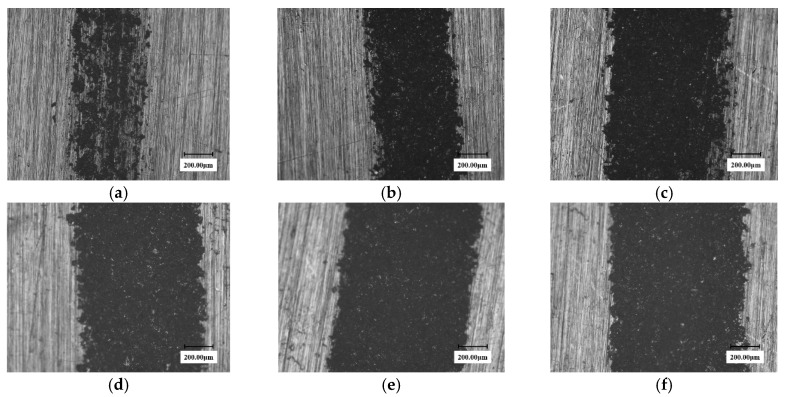
The Surface microscopic morphology of 90° solid solution reinforced samples at different times. (nozzle diameter: 0.2 mm). (**a**) 60 min; (**b**) 120 min; (**c**) 150 min; (**d**) 270 min; (**e**) 330 min; (**f**) 390 min.

Figure 3 and Figure 4 show the surface micro-morphologies of the substrate and the solid solution reinforced samples, respectively, at different test times under 0.2 mm nozzle diameter conditions. It can be seen from the figures that the erosion width of the solid solution sample at any test time is smaller than that of the substrate sample.

**Figure 5:**
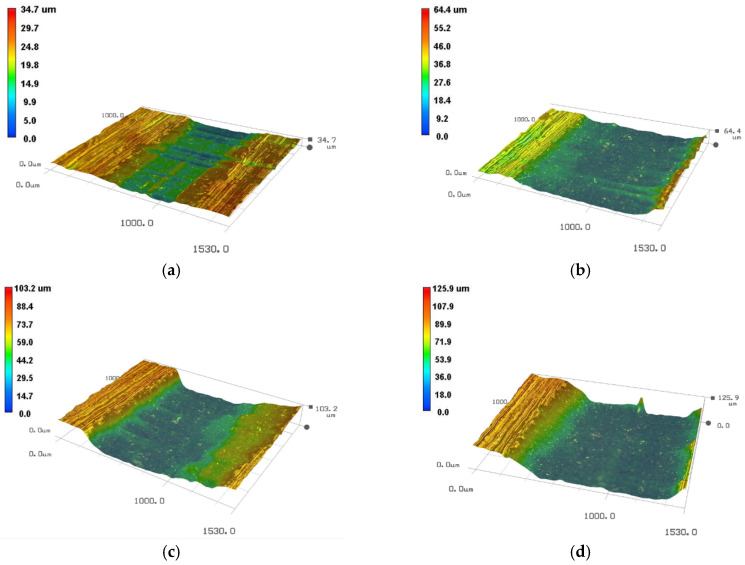
The 3D pit topographies of substrate 90° sample at different time. (**a**) 150 min; (**b**) 270 min; (**c**) 330 min; (**d**) 390 min.

**Figure 6:**
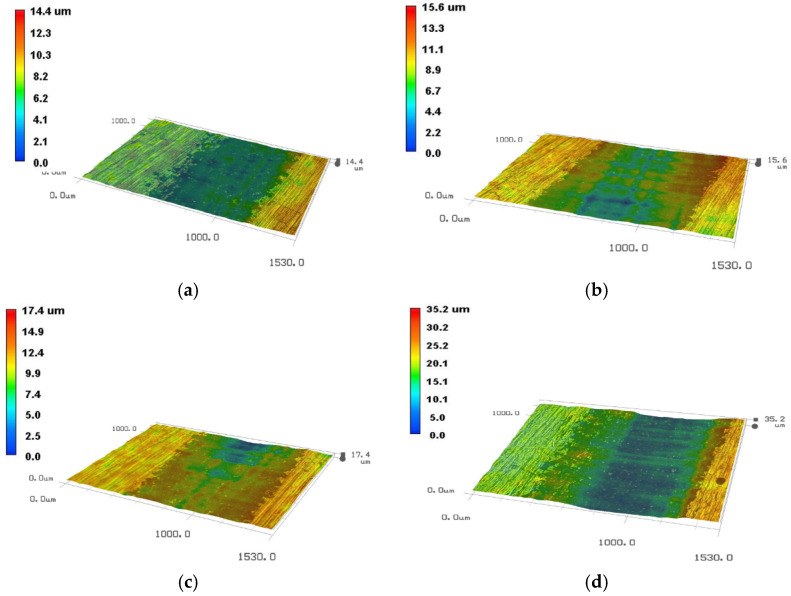
The micromorphologies of the solid solution enhancement 90° sample at different times. (**a**) 150 min; (**b**) 270 min; (**c**) 330 min; (**d**) 390 min.

Since the 2D microscopic morphologies cannot reflect the depth of water droplet erosion grooves, the 3D pit topographies of martensite substrate and solid solution reinforced sample obtained at four typical moments using the VHX-600 are presented in Figure 5 and Figure 6. It can be clearly seen that the groove depth of the solid solution enhanced sample is obviously shallower than that of substrate at the same time.

**Figure 7:**
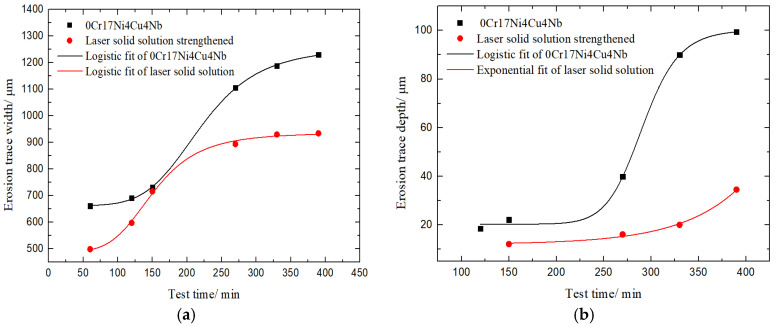
Curves of impact width and depth at different time of substrate and solid solution reinforced samples. (**a**). The erosion trace width along test time; (**b**) The erosion trace depth along test time.

In order to further quantitatively compare the WDE characteristics of these two samples, the average width and depth of water droplet erosion traces of martensite substrate and solid solution samples at different times are fitted into logistic and exponential curves, as shown in Figure 7. The accuracy of these fitted curves is shown in Table 2. All Adj. R-Squares are above 0.9, indicating the high fitting accuracy. As Figure 7 shows, the erosion traces development is relatively consistent. When the surface is broken, the width and depth increase dramatically. Due to the limitation of the jet diameter, the width increase gradually becomes slow down, but both width and depth of the solid solution strengthened sample are less than that of the martensite material. The water droplet erosion resistance is obviously improved after the treatment of solid solution strengthening.

**Figure 8:**
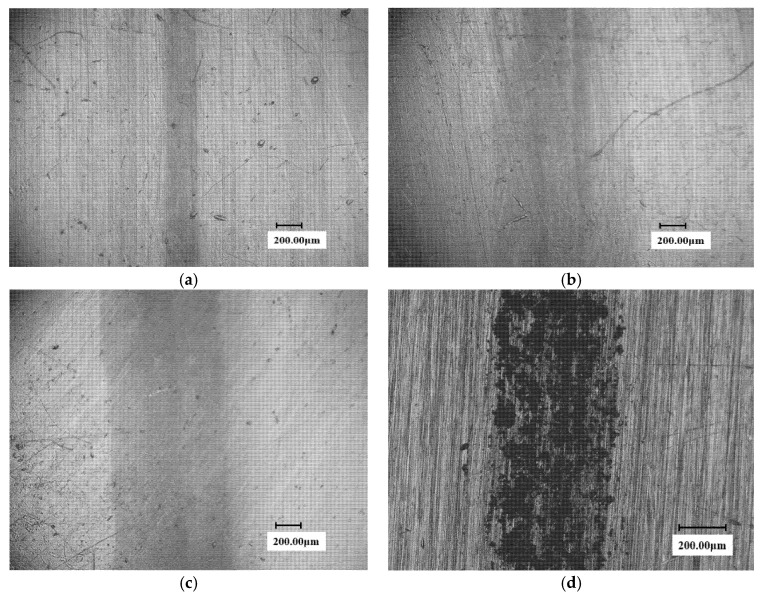
Surface micromorphology of cladding samples at different time. (**a**) 30° sample (erosion trace width: 290 μm); (**b**) 60° sample (erosion trace width: 600 μm); (**c**) 90° sample (erosion trace width: 800 μm); (**d**) solid solution strengthened 90° sample 60 min (erosion trace width: 660 μm).

Since laser cladding forms a denser cladding layer on the substrate surface, it theoretically has better WDE resistance. Figure 8 shows the eroded surface micromorphologies of three angle laser cladding samples after 450 min.

As can be seen from the figure, although the test time is longer than that of the substrate and the solid solution samples, no obvious erosion pitting is formed on the surface of the three samples, with only a light-colored trace. The trace widths at different angles are slightly different. The impact mark width of 30° sample was only 290 microns, and the widest impact trace of 90° sample was about 800 microns. In contrast, the solid solution sample formed a very distinct crater at 60 min, which was close to forming a continuous groove.

Combined with the micromorphology of the laser cladding and solid solution samples at various moments, it can be seen that different surface treatment process has a greater effect on the WDE resistance. The sample with laser cladding treatment has much better water droplet erosion resistance than that of the solid solution strengthened sample.

**Figure 9:**
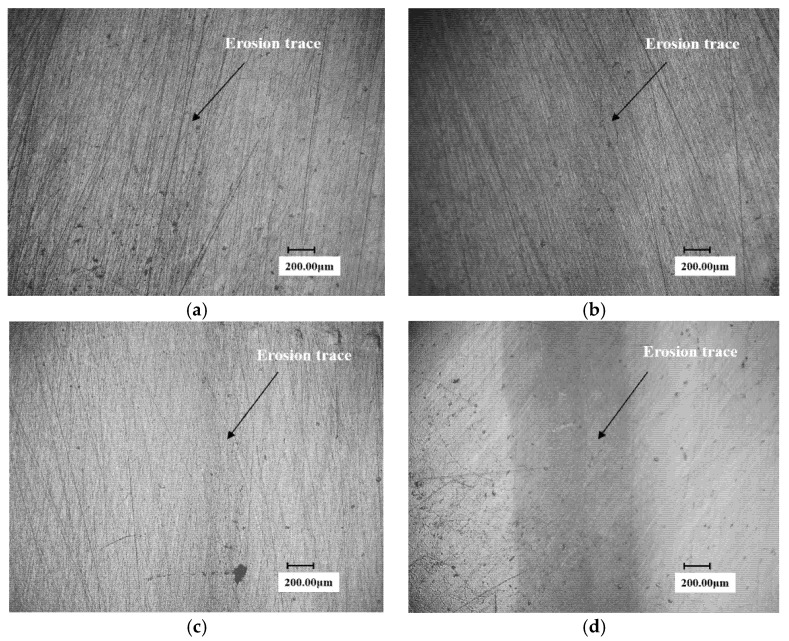
The surface micrograph of stellite alloy specimen at 450 min. (**a**) 30° sample (erosion trace width: 200 μm); (**b**) 60° sample (erosion trace width: 380 μm); (**c**) 90°sample (erosion trace width: 400 μm); (**d**) laser cladding 90° sample (erosion trace width: 800 μm).

The brazed stellite sample exhibited excellent WDE resistance performance. During the experiment, the surface of the eroded samples still almost kept intact after a long period of test time. Under the visual observation, only the color of the WDE zone on the sample surface was found to be darkened and blackened without any distinguishable damage features. Figure 9 shows the eroded micromorphology of the stellite alloy surface at 450 min.

It can be seen from the figure that only a very shallow erosion trace appeared on the surface of the three angles stellite alloy samples and the trace of the 30° sample is very light and could hardly be distinguished. The erosion width of the 90° sample is also slight and is only about 400 microns. The trace of the laser cladding sample is very evident and is about 800 microns in size. Therefore, it is not difficult to deduce that the WDE resistance of the brazed stellite alloy is significantly superior to the laser cladding material.

**Figure 10:**
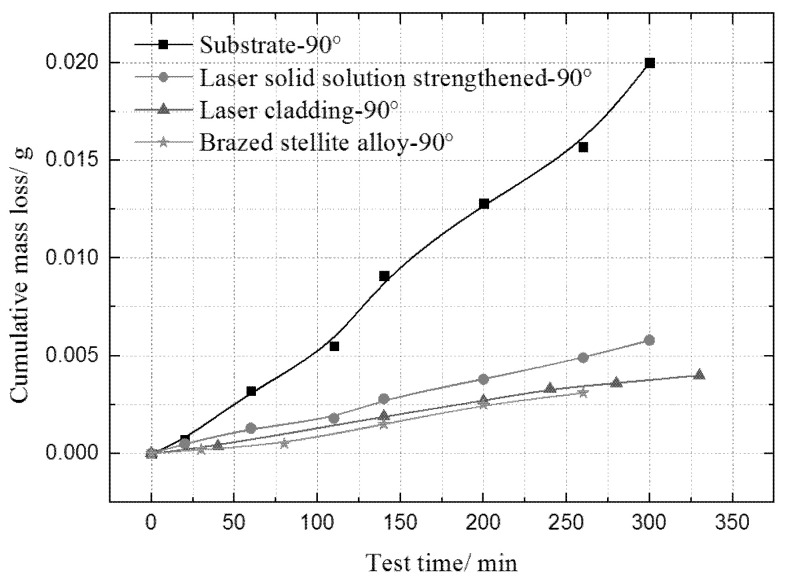
The comparison of mass loss of four different samples at 90°.

In order to quantitatively investigate the water droplet erosion process, the mass loss of the 0Cr17Ni4Cu4Nb martensitic substrate, solid solution, cladding and brazed stellite alloy specimens were compared under the condition of 0.2 mm diameter waterjet nozzle, as shown in Figure 10. It can be seen from the figure that the mass loss of stellite alloy and laser cladding is the smallest. Thus they show the most excellent WDE resistance performance. The martensitic substrate has the worst WDE resistance. The WDE resistance of samples after solid solution strengthening is significantly improved compared with the martensitic substrate. From the mechanical properties of the samples (see Table 3), it is found that the micro Vickers hardness values of the four samples have not changed distinctly. Even the hardness of the cladding samples is slightly higher than that of the brazed stellite alloy, it can be seen that the WDE resistance of brazed stellite alloy is much higher than that of solid solution samples, and slightly better than that of cladding samples. Moreover, in the sample machining process, stellite alloy plate was directly welded on the substrate. During the erosion process, the waterjet swept to the welding edge, it would erode the welding part. However, the WDE resistance of the welding part is inferior to the brazed stellite alloy. The mass loss error of the brazed stellite alloy will be relatively larger. Even so, the mass loss of brazed stellite alloy is still the smallest, which exhibits the optimal WDE resistance. The result further indicates that hardness is not the decisive factor for the WDE resistance of brazed stellite alloy.

Many researchers have tried to obtain the intrinsic relationship between the WDE resistance of materials and mechanical properties such as hardness and fracture strain energy, but unfortunately all these attempts have failed. For example, at the same hardness level, the WDE resistance of stellite alloy is much higher than that of austenitic and martensitic stainless steels [19]. The fracture strain energy of alloy 718 was five times higher than that of stellite 6B alloy, but its WDE resistance was two times smaller than that of stellite 6B alloy [20]. According to the hardness test, the hardness of 12% Cr martensitic steel and stellite 6B alloy are 380 kg/mm^2^ and 420 kg/mm^2^, respectively, which are basically at the same level. However, the water droplet erosion resistance of stellite 6B alloy was 6 times more than that of 12% Cr martensitic steel [21]. These results also fully indicate that hardness is not the decisive factor of the WDE resistance.

**Figure 11:**
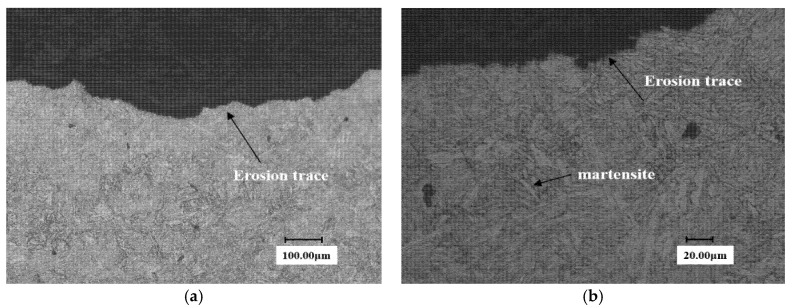
Metallographic diagram of substrate surface. (**a**) Close to end face of the erosion trace (×300) (**b**) Close to end face of the erosion trace (×1.0 k).

In view of this, the authors have carried out a metallographic analysis for the samples after the WDE tests. Figure 11 is the metallographic diagram of substrate surface under two different magnifications. It can be seen that the grain profile is clear and presents a lath martensite structure for martensite substrate.

**Figure 12:**
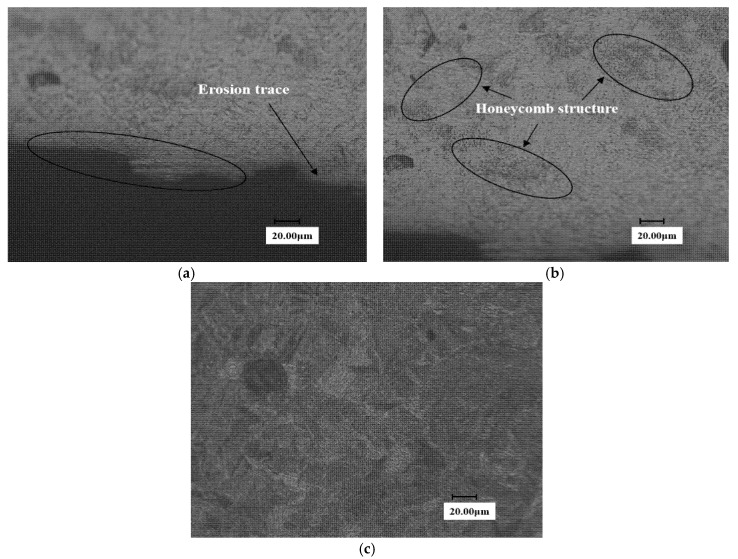
Surface metallographic diagram of solid solution reinforced samples. (**a**) Close to end face of the erosion trace (**b**) transition zone of laser solid solution strengthened sample (**c**) Substrate section of laser solid solution strengthened sample.

The sample after solid solution strengthening is shown in Figure 12. And a high-density area is formed in the solid solution zone, as shown in Figure 12a. It is preliminarily judged that the refined low-carbon martensite has a higher dislocation density, which improves the WDE resistance of the material to a certain extent. However, compared with the solid solution transition region and the substrate, it can be found that there is a honeycomb-like morphology in the solid solution strengthening transition region (see Figure 12b), which may limit the further improvement of erosion resistance.

**Figure 13:**
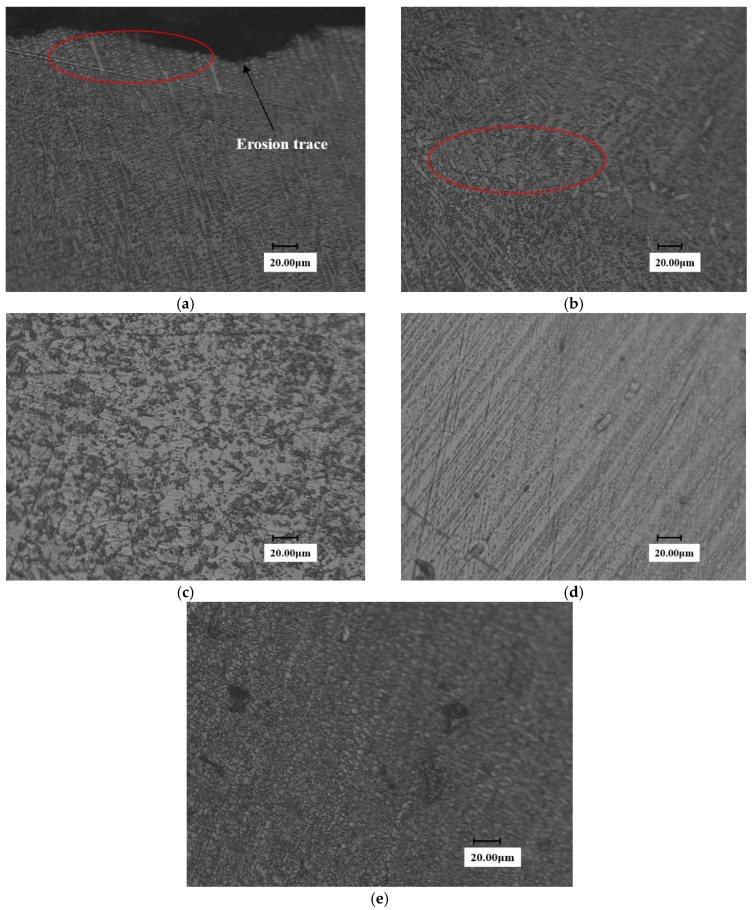
Metallographic diagram of cladding sample surface. (**a**) End face of laser Cladding sample (**b**) Transition zone of laser Cladding sample (**c**) Substrate section of laser Cladding sample (**d**) No erosion zone on the surface (**e**) erosion zone on the surface.

Figure 13 shows the metallographic structure of the eroded surface of the laser cladding sample. It can be seen from Figure 13a that the surface grains after laser cladding are very dense. With the increasing distance from the cladding surface, the grains gradually become loose. And the grains are no longer visible at the substrate region far away from the surface, so the compact structure is just the key that allows materials to avoid excessive concentration of energy when impact by a waterjet, improving the toughness and impact resistance of materials. Figure 13d,e are micrographs of the no WDE area and eroded area, respectively. It can be seen that the grains structures on the surface of the eroded area are similar to those in Figure 13a. It is proved that the WDE damage is slightly in the waterjet process, only the superficial layer of the surface was destroyed. The grains remain dense.

**Figure 14:**
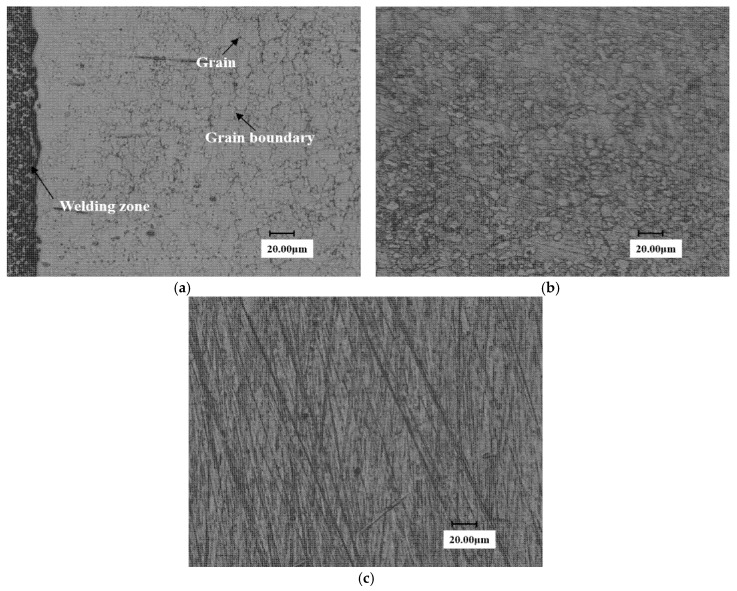
Metallographic diagram of stellite alloy specimen. (**a**) End face of Stellite alloy (**b**) erosion zone on the surface (**c**) No erosion zone on the surface.

Figure 14 shows the metallographic diagram of the eroded stellite alloy. Figure 14a shows the metallographic structure of stellite alloy. Figure 14b,c is the microstructure of eroded area and no WDE area on the 90° sample surface, respectively. 

It can be seen from the figure that grain boundary is faintly visible on the stellite alloy surface and the grain structure is not obvious yet. A possible reason is that the superficial layer of the surface has not been completely destroyed.

**Figure 15:**
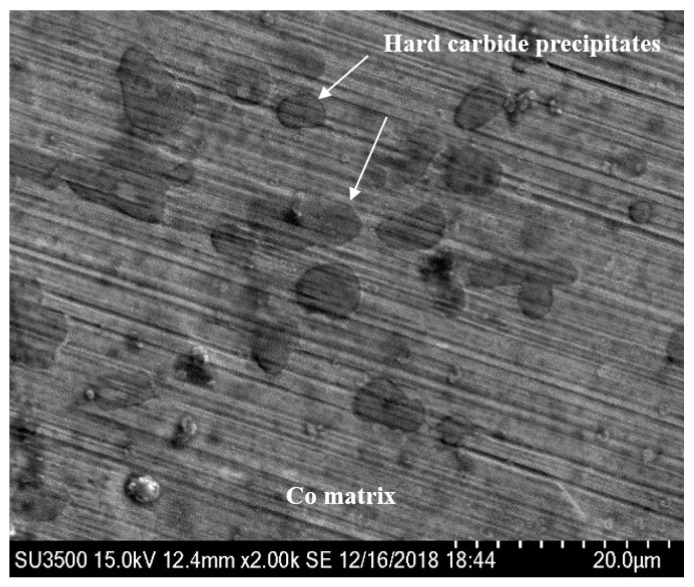
Micromorphology of stellite alloy surface without water droplet erosion.

**Figure 16:**
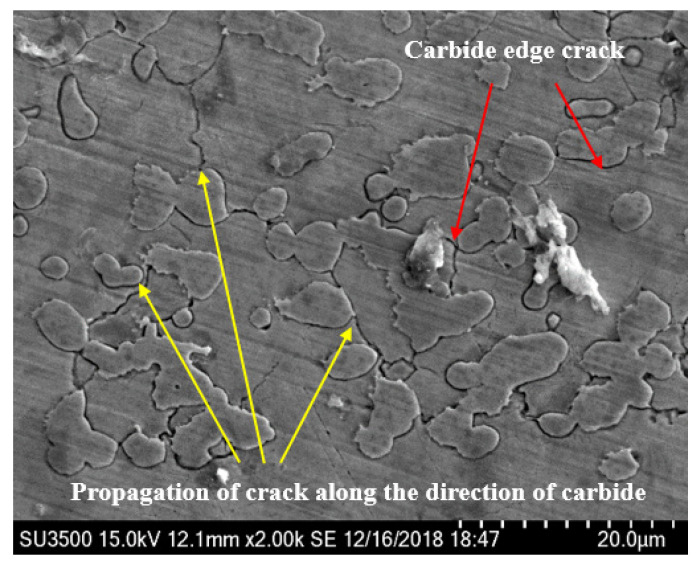
Micromorphology of water droplet erosion zone of stellite alloy. (×2.0 k).

**Figure 17:**
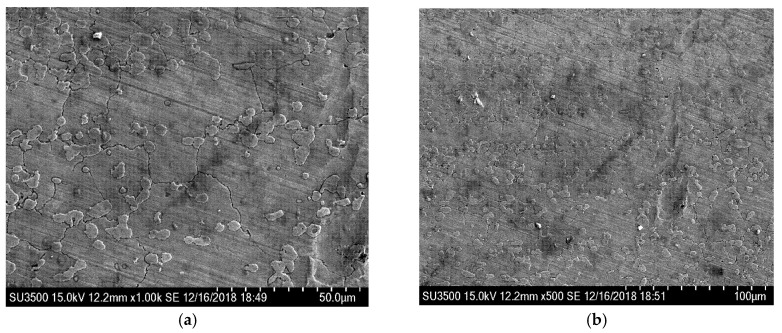
Micromorphology of water droplet erosion zone in stellite alloy. (**a**) ×1.0 k; (**b**) ×500.

In order to further analyze the deep reason for the excellent WDE resistance of the brazed stellite alloy, scanning electron microscope was used to focus on the microscopic observation of the darkening and blackening WDE area after 450 min water droplet erosion test on the brazed stellite alloy sample. The observation results are shown in Figure 15, Figure 16 and Figure 17. From Figure 15, it can be seen that the stellite alloy consists of a cobalt-based solid solution main phase and the reinforced carbide precipitates secondary phase with shape edges.

Combined with Figure 16 and Figure 17, it can be seen that there is almost no plastic deformation on the stellite alloy surface and little material removal on the cobalt matrix. Thomas [17] also found that the cobalt alloy had the best WDE resistance in several metals and alloys. And the fracture strength and strain energy of the cobalt alloy had no advantage in WDE test, but the deformation mode was very unique. In the early stages of water droplet erosion, the target surface did not appear any type of pit. And the impact area formed a very uniform block type. It was not until 150 × 10^3^ times of water droplet impact that the weight loss of the material was detected. The shear and fracture were presented in the most severely deformation area.

In addition, it can be clearly seen from Figure 16 and Figure 17 that the visible darkened and blackened eroded zone of the stellite alloy forms some cracks, which are initiated at the hard carbides boundaries and propagated along the direction of carbides. Under the continuous water droplet erosion, cracks grow and connect into networks, resulting in the removal of carbide precipitates and WDE damage.

Under the impact of waterjet, the microstructure of cobalt matrix will undergo subtle changes. A prominent feature is the formation of mechanical twins. It has been demonstrated that the excellent erosion resistance of stellite alloy is due to the deformation of mechanical twins in the cobalt matrix [22,23,24,25,26]. When they are subjected to impacted, the deformation caused by the transition from a stable face-centered structure to a metastable hexagonal close-packed structure can absorb most of the energy. Preece et al. [27] reported that twins had the effect of dividing the original grains into smaller segments (the size in the range of 0.1 to 1.0 μm). As the number of impacts increases, the increasing twin density will break grains into sub-micron size, which will reduce the mean free path of dislocations, thereby limiting surface deformation and thus higher erosion resistance. A large number of studies reported in the literature also show that as the grain size decreases, the WDE resistance increases [22,25,28,29].

Through a large number of microscopic observations on the surface of the stellite alloy samples, it is found that the cracks in the erosion damage zone are initiated at the interface and the interior of the hard carbide precipitates, resulting the internal stress concentration under the cumulative impact. Then the internal stress concentration points cause the initiation and propagation of cracks along the interface and interior of the hard carbide precipitates. Due to the formation of crack network caused carbides removal and erosion damage. The material loss in stellite alloy is observed mainly at the carbide precipitation site, while the cobalt matrix that is formed mechanical twins was much less damaged than that of the carbides.

It has been reported [30] that the water droplet erosion resistance of cobalt alloy without carbides and stellite 6B alloy was experimentally investigated, and the results also confirmed that the Co alloy was slightly better than carbide-containing stellite 6B alloy. Combined with the above analysis, it is not difficult to determine that the stable face-center structure of cobalt matrix will transform to the metastable hexagonal close-packed structure under impact. And excellent WDE resistance of stellite alloy is mainly related to the deformation and energy absorption involved in this transformation. In addition, in the microscopic observation of the stellite alloy, it was found that the hard carbide in the stellite alloy is the starting point of crack formation and propagation. Under the continuous droplet impact, the formation of crack network causes carbides removal and erosion damage, which is the main reason for the mass loss of stellite alloy. It is proved that the properties of the Co-based material itself is the reason for its excellent WDE resistance, and the carbides have almost no positive contribution to its anti-erodibility. These new findings are of great significance to process methods and parameter selection of steam turbine blade materials and surface strengthened layers.

## 4. Conclusions

Based on the high-speed waterjet test system, the WDE performances of the typical martensite blade substrate 0Cr17Ni4Cu4Nb and surface strengthening materials: laser solution strengthening samples, stellite laser cladding samples and brazed stellite alloy samples are investigated. The influence of the surface strengthened process on the water-erosion characteristics is investigated systematically. The main conclusions are as follows:

1) After solid solution strengthening, the sample hardness has been increased and the WDE resistance has been significantly improved. However, according to the metallographic analysis, there is a suspected honeycomb defect in the strengthened region, which may limit the further improvement of the WDE resistance.
2) At the same impact angle, the WDE resistance characteristics of martensite substrate, solution strengthening, laser cladding and stellite alloy samples are in order from excellent to inferior: brazed stellite alloy, laser cladding, solid solution strengthening and martensite substrate. Laser cladding and stellite alloy materials have excellent WDE resistance. Combined with the micro Vickers hardness test, it is fully indicated that the hardness parameter is not the decisive factor of WDE resistance of brazed stellite alloy.
3) Furthermore, the WDE resistance mechanism and the failure mode of brazed stellite alloy have been revealed. It is found that the cracks in the erosion damage zone are initiated at the interior and boundary of the hard carbide precipitates. And the cracks expanded along the direction of the carbides. Under the continuous droplet impact, cracks grow and connect into networks, resulting in the removal of carbide precipitates and WDE damage. It is proved that the properties of the Co-based material itself is the reason for its excellent WDE resistance, and the carbides have almost no positive contribution to its anti-erodibility.

## Figures and Tables

**Table 1:** Comparison parameters of material resistance to WDE.

Samples	*V*_0_/m·s^−1^	*θ*/°	Nozzle Diameter/mm	Number of the Sample
0Cr17Ni4Cu4Nb	650	30	0.15	≤6
		60	0.15	≤6
		90	0.15, 0.2	≤6
laser solid solution sample	650	90	0.15, 0.2	≤6
stellite laser cladding sample	650	90	0.15, 0.2	≤6
brazed stellite alloy sample	650	30	0.15	≤6
		60	0.15	≤6
		90	0.15, 0.2	≤6

**Table 2:** The accuracy of these fitted curves.

Materials	Adj. R-Square (Width)	Adj. R-Square (Depth)
0Cr17Ni4Cu4Nb	0.9996	0.99556
Laser solid solution	0.99527	0.98791

**Table 3:** The mechanical parameters of several samples.

Materials	*T*_D_/μm	*H*/HV	*σ*_b_/MPa	*G*_T_/GPa
0Cr17Ni4Cu4Nb	-	330~350	890	77.3
Laser solid solution	250~300	400	1290	-
Laser cladding	250~300	440	1414	-
Brazed stellite	-	380	694	97

## References

[1] Hesketh J., Walker P.J. (2005). Effects of Wetness in Steam Turbines. Proc. Inst. Mech. Eng. Part C J. Mech. Eng. Sci..

[2] Crane R. (2004). Droplet deposition in steam turbines. Proc. Inst. Mech. Eng. Part C J. Mech. Eng. Sci..

[3] Heymann F. (1992). Liquid Impingement Erosion.

[4] Gardner G.C. (1963). Events Leading to Erosion in the Steam Turbine. Proc. Inst. Mech. Eng..

[5] Carter T.J. (2005). Common failures in gas turbine blades. Eng. Fail. Anal..

[6] Mahdipoor M., Kirols H., Kevorkov D., Jedrzejowski P., Medraj M. (2015). Influence of impact speed on water droplet erosion of TiAl compared with Ti6Al4V. Sci. Rep..

[7] Kirols H., Kevorkov D., Uihlein A., Medraj M. (2015). The effect of initial surface roughness on water droplet erosion behaviour. Wear.

[8] Mahdipoor M., Tarasi F., Moreau C., Dolatabadi A., Medraj M. (2015). HVOF sprayed coatings of nano-agglomerated tungsten-carbide/cobalt powders for water droplet erosion application. Wear.

[9] Tarasi F., Mahdipoor M.S., Dolatabadi A., Medraj M., Moreau C. (2016). HVOF and HVAF Coatings of Agglomerated Tungsten Carbide-Cobalt Powders for Water Droplet Erosion Application. J. Therm. Spray Technol..

[10] Smith A., Caldwell J., Pearson D., McAllister D.H., Christie D.G. (1966). A discussion on deformation of solids by the impact of liquids, and its relation to rain damage in aircraft and missiles, to blade erosion in steam turbines, and to cavitation erosion—Physical aspects of blade erosion by wet steam in turbines. Philos. Trans. R. Soc. London. Ser. A, Math. Phys. Sci..

[11] Hattori S., Kakuichi M. (2013). Effect of impact angle on liquid droplet impingement erosion. Wear.

[12] Xie Y.-H., Wang Y., Chen J.-H., Zhang D. (2009). Numerical and Experimental Study on Mechanism of Material Damage by High Speed Liquid-solid Impact. Proc. CSEE.

[13] Xu W., Wang J., Qin L., Chen H., Chen D. (2010). Investigation of Erosion Damages Induced by Wet Steam Containing Micro-Particles. Tribol. Lett..

[14] Wanli X., Li Q., Haosheng C., Darong C. (2010). Erosion and abrasion on mild carbon steel surface by steam containing SiC microparticles. Wear.

[15] Xu W.L. (2010). Research on Mechanism of Erosion Induced by Wet Steam. Ph.D. Thesis.

[16] Oka Y., Miyata H. (2009). Erosion behaviour of ceramic bulk and coating materials caused by water droplet impingement. Wear.

[17] Thomas G.P., Brunton J.H. (1970). Drop impingement erosion of metals. Proc. Roy. Soc. London. Ser. A.

[18] Ahmad M., Casey M., Sürken N. (2009). Experimental assessment of droplet impact erosion resistance of steam turbine blade materials. Wear.

[19] Heymann F. (1970). Toward Quantitative Prediction of Liquid Impact Erosion. Characterization and Determination of Erosion Resistance.

[20] Antony K.C. The effect of composition and microstructure on cavitation erosion resistance. Proceedings of the 5th International Conference on Erosion by Liquid and Solid Impact.

[21] Jeong C.K., Kim W.W., Rhee C.K., Lee W.J. (1998). Investigation of liquid impact erosion for 12Cr steel and Stellite 6B. J. Nucl. Mater..

[22] Graham A.H., Youngblood J.L. (1970). Work strengthening by a deformation-induced phase transformation in “MP alloys”. Met. Mater. Trans. A.

[23] Woodford D.A. (1972). Cavitation-erosion-lnduced phase transformations in alloys. Met. Mater. Trans. A.

[24] Rémy L., Pineau A. (1976). Twinning and strain-induced F.C.C. → H.C.P. transformation on the mechanical properties of Co-Ni-Cr-Mo alloys. Mater. Sci. Eng..

[25] Vaidya S., Mahajan S., Preece C.M. (1980). The role of twinning in the cavitation erosion of cobalt single crystals. Met. Mater. Trans. A.

[26] Heathcock C., Ball A., Protheroe B. (1981). Cavitation erosion of cobalt-based Stellite^®^ alloys, cemented carbides and surface-treated low alloy steels. Wear.

[27] Preece C., Vaidya S., Dakshinamoorthy S. (1979). Influence of Crystal Structure on the Failure Mode of Metals by Cavitation Erosion. Erosion: Prevention and Useful Applications.

[28] Mousson J. (1937). Pitting resistance of metals under cavitation conditions. ASME TRANS.

[29] Robinson M.J., Hammitt F.G. (1967). Detailed Damage Characteristics in a Cavitating Venturi. J. Basic Eng..

[30] Preece C.M. (1979). Treatise on Materials Science and Technology.

